# Progression of lymphatic filariasis antigenaemia and microfilaraemia over 4.5 years in antigen-positive individuals, Samoa 2019-2023

**DOI:** 10.1016/j.ijid.2025.107891

**Published:** 2025-06

**Authors:** Helen J. Mayfield, Benn Sartorius, Ramona Muttucumaru, Sarah Sheridan, Maddison Howlett, Beatris Mario Martin, Shannon M. Hedtke, Emma Field, Robert Thomsen, Satupaitea Viali, Patricia M. Graves, Colleen L. Lau

**Affiliations:** 1 University of Queensland Centre for Clinical Research, Faculty of Health, Medicine, and Behavioural Sciences, The University of Queensland, Brisbane, Queensland, Australia; 2 School of Public Health, Faculty of Health, Medicine, and Behavioural Sciences, The University of Queensland, Brisbane, Queensland, Australia; 3 National Centre for Epidemiology and Population Health, The Australian National University, Canberra, Australia; 4 Department of Environment and Genetics, La Trobe University, Bundoora, Victoria, Australia; 5 Samoa Ministry of Health, Apia, Samoa; 6 Oceania University of Medicine Samoa, Apia, Samoa; 7 College of Public Health, Medical and Veterinary Sciences, James Cook University, Queensland, Australia

**Keywords:** Recrudescence, Neglected Tropical Disease elimination, *Wuchereria bancrofti*, Pacific Islands, Clustering

## Abstract

**Objectives:**

The first round of triple-drug mass drug administration (MDA) for lymphatic filariasis (LF) in Samoa was in 2018. This study aims to i) examine progression of LF antigen (Ag) and microfilaria (Mf) in Ag-positive individuals from 2019-2023; and ii) compare Ag/Mf prevalence in household members of Mf-positive vs Mf-negative participants.

**Methods:**

In 2023, we tested Ag-positive participants (indexes) from a 2019 survey in Samoa, and their household members. We tested for Ag (Alere/Abbott Filariasis Test Strip) and Mf. We examined changes in Ag/Mf status in index participants and compared Ag/Mf prevalence between household members of Mf-positive and Mf-negative indexes.

**Results:**

We recruited 91 indexes and 317 household members. In 2023, all 17 Mf-positive indexes remained Ag-positive and 11/15 with Mf results (73.3%) were Mf-positive. Of 74 Mf-negative indexes, 79.7% remained Ag-positive in 2023 and 31.1% became Mf-positive. Household members of Mf-positive indexes were more likely to be Ag-positive (odds ratios 3.3, 95% CI 1.0-10.3) compared to those of Mf-negative indexes.

**Conclusion:**

Our results raise concerns regarding long-term effectiveness of a single-dose of triple-drug MDA for sustained clearance of Mf in Samoa. Guidelines for follow-up and treatment of Ag/Mf-positive people and household members are urgently required.

## Highlights

• Persistent microfilaraemia in Samoa 4.5-years post-mass drug administration (MDA).
• Single round of triple-drug MDA was not sufficient for sustained clearance of Mf.
• Higher antigen (Ag) prevalence in household members of Mf-positive people.
• Household members of Ag/Mf-positive people should be tested and treated.

## Introduction

Lymphatic filariasis (LF) is a widely endemic vector-borne parasitic disease [1]. If infections are left untreated, the pathogen can damage the lymphatic system resulting in severe, irreversible lymphedema. Considered by the World Health Organization (WHO) as a Neglected Tropical Disease [2], coordinated efforts are being made by the WHO's Global Program to Eliminate LF to eliminate the disease as a public health problem through breaking the parasite transmission cycle between humans and vectors, and managing morbidity in those people already affected. Elimination is validated based on transmission assessment surveys (TAS) of children aged 6-7 years. For a country to pass TAS, the number of antigen (Ag)-positive children must be below a given target threshold, calculated such that the likelihood of an evaluation unit passing is at least 75% if true Ag prevalence is 0.5% and no more than a 5% chance of passing if the true Ag prevalence is ≥1% [3]. In the Pacific region, the program [4] has been successful in achieving validation of elimination in eight of 16 endemic countries [5]. However, continued transmission remains in some countries, including in Samoa [6], despite decades of elimination efforts.

The key intervention employed by elimination programs is delivery of mass drug administration (MDA) to at-risk populations, aimed at breaking the parasite's transmission cycle. In 2018, WHO recommended treating at-risk populations with a triple-drug regimen of annual rounds of ivermectin, diethylcarbamazine and albendazole (IDA) in areas without endemic onchocerciasis and where two-drug combinations have proven unsuccessful [7]. Several LF-endemic Pacific nations fell into this category, and in August 2018, Samoa was the first country in the world to implement a national triple-drug MDA [8].

The 2018 MDA in Samoa was carried out from 14 to 26 August, 2018 and achieved self-reported coverage of 80% of the total population [9], exceeding the minimum recommendation of 65% by WHO [10]. Given the existing evidence that IDA is effective at immediate clearance (within 30 days) of circulating microfilaria (Mf) in Samoa [11], it was therefore surprising to detect 18 Mf-positive participants from the 121 Ag-positive participants (from 3883 valid Ag tests) who had slides available (overall Mf prevalence of 0.46%) during a 2018 community survey conducted 1-3 months after the completion of the MDA [12]. Although some Mf-positive participants might have incorrectly reported that they ingested the full dose of IDA, it is highly possible that the triple-drug regimen only provides short-term clearance of Mf, which may reappear over the following months and years.

The second round of triple-drug MDA in Samoa was planned for late 2019 but was delayed until September 2023 because of a severe measles outbreak [13] and the COVID-19 pandemic (a timeline for the surveys in relation to the MDA rounds is provided in Supplementary Figure 1). This delay resulted in the loss of any long-term gain from the 2018 MDA, with no significant change detected in Ag or Mf prevalence from 2018 to 2023, based on results from eight primary sampling units (PSUs) [14]. However, the long-term effectiveness of one round of triple-drug MDA in Ag-positive participants at an individual-level has not been quantified. Here, we report on findings from a follow-up survey conducted in 2023, prior to the second round of triple-drug MDA, which re-tested participants who were Ag-positive in 2019. The objectives of this study were to examine i) the long-term (4.5 years) progression of Ag, and Mf, between 2019 and 2023 in participants who had not received further treatment after the 2018 round of MDA (index participants); and ii) to compare Ag and Mf prevalence in household members of Mf-positive vs Mf-negative index participants.

## Methods

### Study setting

The study was conducted in villages across Upolu, Savai'i and Manono Island in Samoa, a Pacific Island nation of around 200,000 people [15]. The majority of the population of Samoa live in predominately rural settings, with urban centres around the capital of Apia on Upolu island, and near Salelolonga on Savai'i. The country is divided into four administrative regions: Apia Urban Area (AUA), North West Upolu, Rest of Upolu (ROU) and Savai'i (SAV). The primary vector for LF in Samoa is *Aedes polynesiensis,* which transmits the *Wuchereria bancrofti* parasite [16].

### Survey design

Prior to this study, a national-level survey was carried out in 2019, 7-9 months after the completion of the triple-drug MDA, as part of the Surveillance and Monitoring to Eliminate LF in Samoa (SaMELFS) project [17,18]. The survey covered 35 PSUs, each consisting of one or two villages. Thirty of the PSUs were randomly selected, and the other five were purposively selected as suspected LF hotspots based on previous surveys. The 2019 survey included a community survey of 15 households per PSU (participants aged ≥5 years) and a convenience survey of 5-9-year-old children. In five high-prevalence PSUs identified from a 2018 survey, additional participants were also enrolled in a targeted survey component, surveying households considered high-risk based on an ensemble model combining geo-statistics and machine learning, similar to that used in Mayfield et al. [19].

The 2023 survey was conducted between the 3rd of March and the 1st of April 2023 (Supplementary Figure 1) and aimed to locate and test Ag-positive participants (index participants) from the 2019 survey. Local field teams attempted to contact participants by phone and by visiting the village where they lived. Household members were also invited to participate if they were aged ≥5 years and resided (or recently resided) in the same household as an index participant. In 2023, any participants who were found to be Mf-positive were offered treatment with IDA.

Participants were divided into four groups for analysis, based on whether they were an Mf-positive index participant from 2019, or lived in the same household as one. Each participant was allocated to only one group: i) Mf-positive index participants; ii) Mf-negative index participants, iii) household members of Mf-positive index participants; and iv) all other household members, i.e., household members of Mf-negative index participants who were not also in the same household as a known Mf-positive index. It should be noted that in many cases, some household members did not participate in the survey, and it is possible that some of these untested household members may have been Mf-positive. In households with two or more index participants, groups were allocated based on the presence of an Mf-positive index. If any of the index participants from multi-index households were Mf-positive in 2019, the Mf-positive index participant was assigned to Group A, and the Mf-negative index participants were considered to be household members of the Mf-positive index participant (i.e., Group C). If none of the index participants in a multi-index household were Mf-positive in 2019, all index participants from the household were included in Group B and were not considered a household member of the other index (i.e., they were not included in Group D). For transparency, Group C was categorised into C1 for those originally enrolled as household members and C2 for the reallocated index participants.

### Sample collection and processing

Participants had a finger prick blood sample collected (up to 500 µL) into a heparin microtainer. Although the *W. bancrofti* parasite is diurnally sub-periodic, and we would not expect collection time to substantially affect Mf density, some variation could be expected. Samples were therefore collected between 2 pm and 8 pm, which is estimated to be the peak time for *W. bancrofti* Mf in the Samoan Islands [20]. Once collected, samples were kept in cooler bags while transported to the field laboratory, where they were stored at 4° Celsius and tested within 48 hours. Samples were tested for the presence of LF Ag using Alere/Abbott Filariasis Tests Strips (FTS) (Scarborough, ME, USA), read at 10 minutes. Invalid tests were repeated if there was sufficient sample available.

Three slides were prepared for each Ag-positive sample, two of which were stained for reading, and one left unstained as a backup. One stained slide was initially read in Samoa to establish the presence or absence of Mf so that Mf-positive people could be prioritised for treatment. Both stained slides were then read in Australia where a full Mf count was carried out by two independent readers who each read one slide per Ag-positive sample. For each Mf-positive participant, Mf density per mL was calculated over the two slides.

### Data analysis

Data were analysed using Stata software package version 17.0 (StataCorp, College Station, TX, USA). We calculated descriptive summary statistics for age (median, range) and sex. We used data from the 2019 SaMELFS survey to calculate the proportion of index participants who reported taking MDA in 2018, summarised their Ag/Mf status in 2019 and 2023 and displayed this using a Sankey plot. We then assessed the change in Mf density between 2019 and 2023 among index participants by comparing the geometric mean Mf densities, (calculated by substituting a value of 1 for any zero values) and compared these between groups using the Wilcoxon rank-sum (Mann–Whitney) test.

To examine the prevalence for each of the four groups, we employed a bootstrap method with 1000 replications to estimate Ag and Mf prevalence (and 95% confidence intervals using Agresti method and adjusted for within household clustering) by group. We used a two-level (individuals nested within households) mixed-effects logistic regression model to examine differences in demographics between index participants and their household members. To assess whether the Mf status of index individuals influenced the odds of a household member being Ag-positive and/or Mf-positive, we used the same hierarchical model formulation. Using logistic regression, we examined the odds ratios (OR) of a household member being Ag-positive or Mf-positive in 2023 based on the Mf status of their index case in 2019. Results were adjusted for age, sex and region in the Ag model and gender and age in the Mf model, where region was excluded due to zero Mf-positives in AUA and ROU regions.

To estimate prevalence in the general community, we used data from the 2019 household community survey [17] to calculate Ag and Mf prevalence (adjusted for age, sex and PSU selection probability) in the 22 PSUs where index participants were recruited for this study. The Ag and Mf prevalence in the 22 PSUs in 2019 was considered a reasonable estimate of community prevalence for 2023 based on results of a 2023 survey that showed no significant difference in Ag or Mf prevalence between the 2 years [14] (ref). We compared the Ag and Mf prevalence in the community with that in Groups C and D using prevalence ratios.

## Results

### Participant characteristics

From the 2019 survey, 182 Ag-positive participants with known Mf status were identified as potential index participants; 40 (22.0%) Mf-positive and 142 (78.0%) Mf-negative. A further eight Ag-positive participants from 2019 with unknown Mf status were excluded from this study. Of these 182 people, 74 (40.6%) lived in one of the five purposively selected PSUs, 114 (62.6%) were male and 68 (37.3%) were female. The median age in 2019 was 35 years (range 5-86 years).

**Figure 1:**
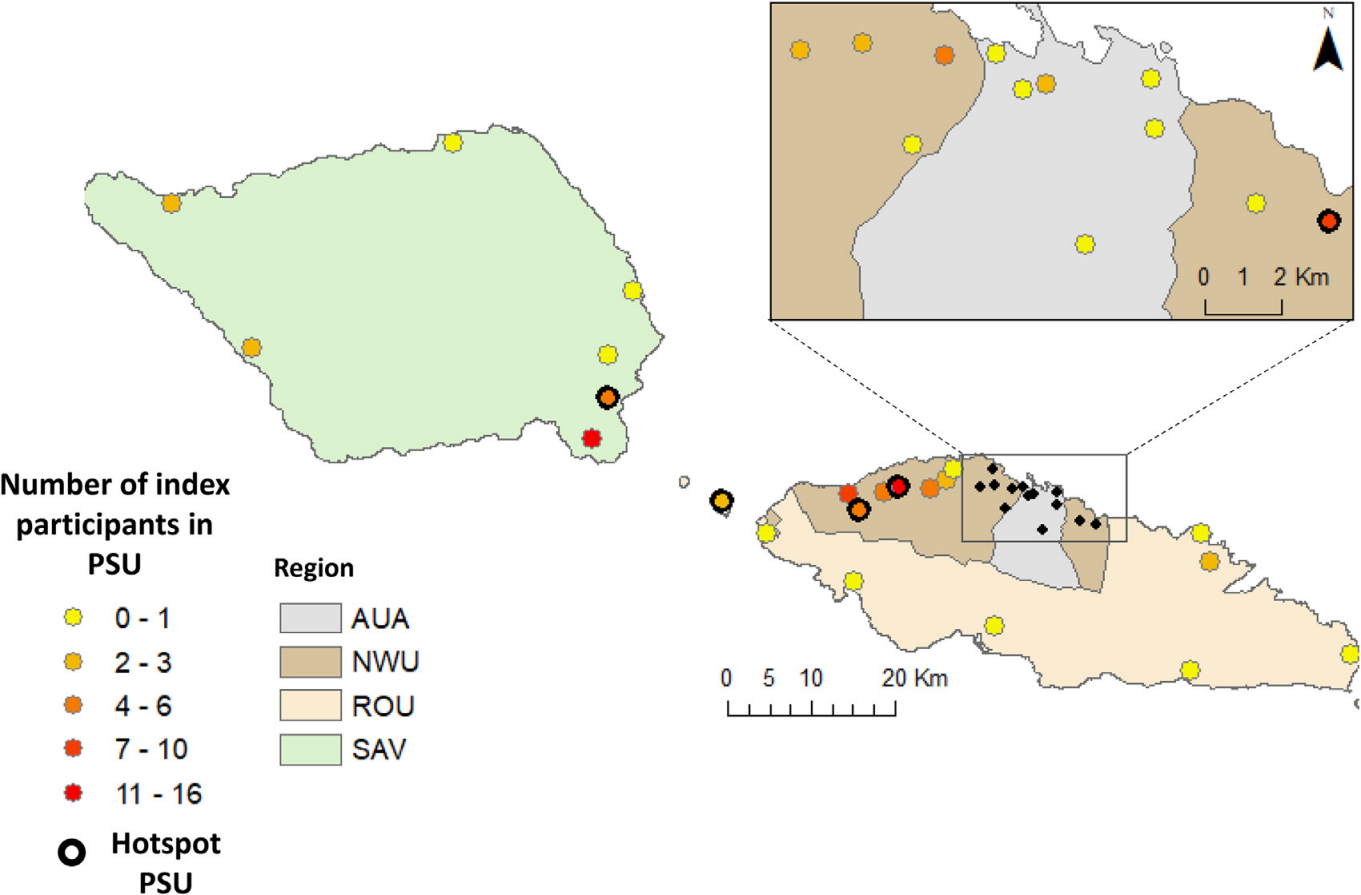
PSU location and number of index participants enrolled per primary sampling unit (PSU) in the 2023 follow-up study in Samoa in the four regions: Apia Urban Area (AUA), North West Upolu (NWU), Rest of Upolu (ROU) and Savai'i (SAV).

In 2023, we recruited 91 (50.0%) of the 182 potential index participants: 17 (42.5%) of the 40 Mf-positive and 74 (52.1%) of the 142 Mf-negative people. Of the 91 recruited index participants, 41 (45.6%) lived in one of the five purposively selected PSUs (Figure 1), 56 (61.5%) were male and 35 (38.5%) were female. Index participants were recruited from 79 households across 22 PSUs, with 11 households having two index participants and three households having three (Supplementary Table S1). The median age for the index participants was 45 years (range 9-89 years). During the 2019 community survey which took place 7-9 months post-MDA, the majority of the 91 index participants (86 people, 94.5%) reported that they had swallowed the pills administered during the triple-drug MDA in 2018.

**Figure 2:**
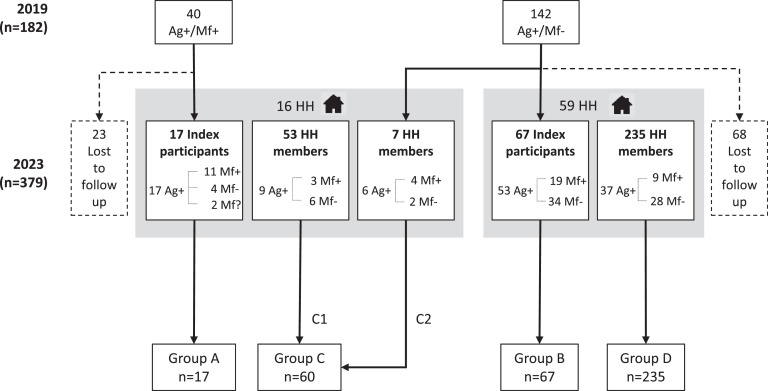
Summary of participants and group allocations for participants in the 2023 follow-up study in Samoa showing numbers of Ag-positive (Ag+), Mf-positive (Mf+) and Mf-negative (Mf–) participants in 2023. For two of the Group A participants, Mf status in 2023 was unknown (Mf?).

Group A consisted of the 17 people recruited from the 2019 Mf-positive participants. There were 74 Mf-negative (in 2019) index participants recruited, 67 of whom were allocated to Group B, and seven who lived in the same household as an Mf-positive index and were allocated to Group C2 (Figure 2, Supplementary Table S2). A total of 53 household members of Group A were allocated to Group C1. Group C therefore consisted of 60 people from 16 households. The remaining 235 participants were household members who were not known to reside with an Mf-positive index participant and were allocated to Group D.

Both index groups (Group A and Group B) had significantly higher odds than their respective household members of being male (Group A vs Group C: OR 10.9 [95% CI 1.1-111.3], *P* = 0.002, and Group B vs Group D: OR 2.4 [95% CI 1.3-4.5], *P* = 0.004) and older (Group A vs Group C: OR 1.1 [95% CI 1.0-1.1 per additional year], *P* = 0.002, and Group B vs Group D: OR 1.1 [95% CI 1.03-1.06], *P* < 0.001). The demographic characteristics of the 379 participants from 20 PSUs across Upolu, Savai'i and Manono Island are outlined in Table 1.

**Table 1:** Index and household participant characteristics in the SaMELFS 2023 follow-up survey in Samoa. Ag-positivity and Mf-positivity refer to the status of the participant in 2019.

		**Index participants**		**Household members**	
		**Group A**	**Group B**	**Group C**	**Group D**
		Mf-positive index in 2019	Mf-negative index in 2019	Household members of Mf-positive index	Household members of Mf-negative index
Total		17	67	60	235
Sex *n* (%)	Male	12 (70%)	41 (61.2%)	20 (33.3%)	99 (42%)
	Female	5 (29%)	26 (38.8%)	40 (66.7%)	136 (58%)
Median age (range)		53 (26-87)	42 (10-89)	18 (5-76)	16 (5-81)
Region *n* (%)	AUA	1	2	14	19
	NWU	11	41	26	168
	ROU	0	9	0	20
	SAV	5	15	20	28
Reported taking MDA in 2018^a^		16 (94%)	63 (94%)	NA	NA

The four regions are: Apia Urban Area (AUA), North West Upolu (NWU), Rest of Upolu (ROU) and Savai'i (SAV).

a Self-reported during the 2019 survey.

### Changes in Ag and Mf status in index participants

**Figure 3:**
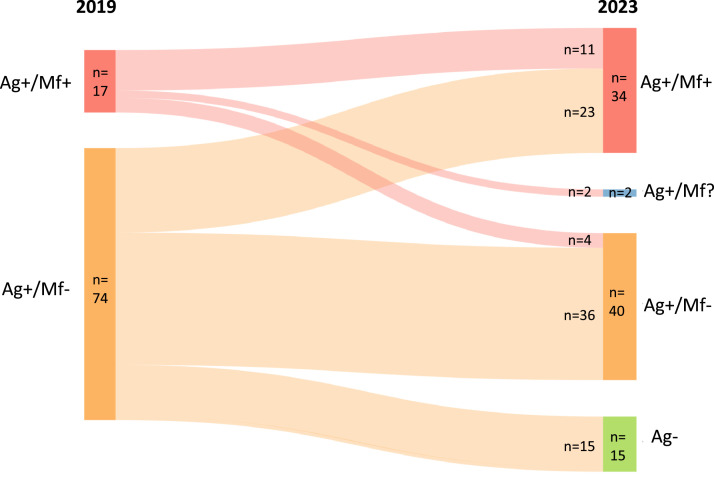
Changes in antigen (Ag) and microfilaria (Mf) status of index participants (*n* = 91) in Samoa between 2019 and 2023. No treatment was given between the 2 years.

All 17 index participants who were Mf-positive in 2019 (Group A) were Ag-positive in 2023. Mf results were not available for two of these participants. Of the remaining 15 Mf-positive index participants, 11 (73.3%, 95% CI 50.5%-95.5%) were Mf-positive in 2023, representing a nonsignificant reduction in Mf prevalence of 26.7% (*P* = 0.125) from 2019. Of the 74 index participants who were Mf-negative in 2019 (Groups B and C2), 59 (79.7%, 95% CI 69.7%-88.3%) remained Ag-positive in 2023. Despite being Mf-negative in 2019, 23 of the 74 participants (31.1%, 95% CI 20.5%-41.5%) were Mf-positive in 2023. Of the 11 index participants in Group A who were Mf-positive in both years, 10 (90.9%) reported during the 2019 survey that they recalled taking the MDA in 2018. Changes in Ag and Mf status of the index cases over the period from 2019 to 2023 are summarised in Figure 3.

For the 15 participants in Group A with Mf results in both years, there was no significant change in geometric mean Mf density from 2019 (201.1 Mf/mL) to 2023 (199.0 Mf/mL) (*P* = 0.281). There were 34 index participants who were Mf-positive in 2023; 11 from Group A and 23 from Groups B and C2. The 11 Mf-positive participants from Group A had a geometric mean Mf density of 333.2 Mf/mL, significantly higher than the participants from Groups B and C2 combined (97.4 Mf/mL, *P* = 0.031).

### Ag and Mf prevalence in household members

**Figure 4:**
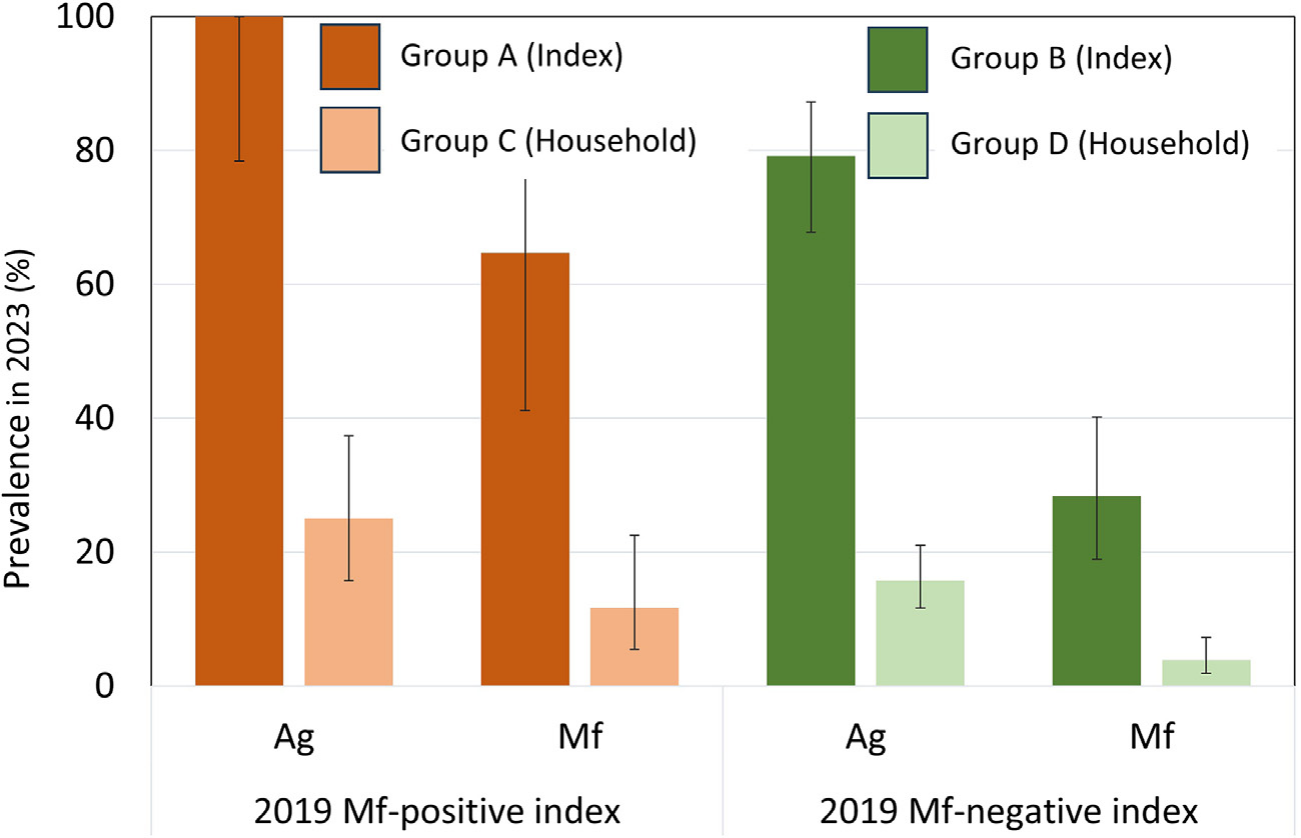
Antigen (Ag) and microfilaria (Mf) prevalence for 2019 Mf-positive (Group A) and Mf-negative (Group B) index participants and their household members in the 2023 SaMELFS follow-up survey. Values are provided in Supplementary Table S4.

Ag and Mf prevalence in household members were significantly lower than for index participants (for Ag: Group A vs Group C: *P* < 0.001, Group B vs Group D: *P* < 0.001; for Mf: Group A vs Group C: *P* = 0.064, Group B vs Group D: *P* = 0.001) (Figure 4 and Supplementary Tables S3 and S4).

Participants in Group C, (who lived with a Group A index participant), had significantly greater odds of being Ag-positive in 2023 compared to those in Group D, who did not live with a Mf-positive index participant (OR 3.27 [95% CI 1.0-10.3], *P* = 0.044). Group C also had higher odds than Group D of being Mf-positive in 2023 (OR 8.5 [95% CI 0.8-97.2], *P* = 0.085), but results were not statistically significant.

Mf-positive index participants (Group A) did not have a significantly higher geometric mean Mf density (*P* = 0.469) compared to their seven Mf-positive household members in Group C (185.3 Mf/mL). Similarly, there was no significant difference (*P* = 0.121) between the geometric mean Mf density of the Mf-positive index participants of Group B (89.4 Mf/mL, *n* = 19), compared to the Mf-positive household members in Group D (262.7 Mf/mL, *n* = 9). Changes in individual Mf densities from 2019 to 2023 are shown in Supplementary Figure S2.

Prevalence in the 22 PSUs from the 2019 survey where index participants were recruited in 2023 (*n* = 1599) was 9.6% (95% CI 7.8-11.7) for Ag and 2.6% (95% CI 1.7-3.4) for Mf. The Ag prevalence ratio for household members compared to the wider community was significantly higher for both Group C (2.6 [95% CI 1.4-4.8]) and Group D (1.6 [95% CI 1.0-2.7]). The Mf prevalence ratio between household members and the community was significantly higher for both Group C (4.5 [95% CIs 1.4-13.1]) and Group D (1.6 [95% CIs 1.0-2.7]).

## Discussion

Our study found high rates of Ag persistence in index participants (85% from 2019 to 2023) after the 2018 MDA, demonstrating that one round of triple-drug MDA was not sufficient for sustained reduction of Ag prevalence to below elimination thresholds [21]. Mf persistence among Mf-positive index participants from 2019 to 2023 was also high (64.7%), and the lack of a decrease in the geometric mean density of Mf indicates ongoing Mf production by adult worms. Similarly, there was high Mf prevalence (28.4%) in 2023 in the index participants who were Ag-positive but Mf-negative in 2019, compared to their household members. Amongst the household members, we found a higher Ag prevalence in those who lived with an Mf-positive index participant compared to those who did not.

One-third of the 17 index participants who were Mf-positive in 2019 (Group A) reverted to being Mf-negative in 2023. During the 4.5-year gap between our 2019 and 2023 surveys, it is extremely unlikely that these people received any anti-filarial medications as no treatment programs were running in Samoa during this time. The most likely explanation for the reversion is that the infection had run its course with the adult worms no longer producing Mf, but resulting in persistent residual Ag.

The remaining two-thirds of the Group A index participants who were Mf-positive in both 2019 and 2023 represent an unexpectedly high number of people remaining infected and infectious. Persistence of microfilaraemia is of particular concern because these individuals remained potential sources for onward transmission during the 4.5 years. The life span of adult *W. bancrofti* worms (without treatment) is usually estimated at 6-8 years [22], but empirical evidence of this is limited. Vanamail et al. [23] estimated the fecund life span of *W. bancrofti* at 5-5.4 years, although occasional evidence of much longer life spans (40 years) have been reported [24]. If the fecund life span of adult worms is about 5 years, we could hypothesise that 20% of infected persons would clear their Mf every year, even if no treatment was given (assuming that the person does not have multiple pairs of mating worms of different ages). That this rate of decline was not observed in the current study implies that some of the Mf-positives identified in 2023 were the result of ongoing transmission, or that the maximum lifespan of the adult worms exceeds the current estimates.

There are several aspects of these results that suggest recrudescence was more likely than reinfection as the explanation for the persistent microfilaraemia in over 70% of our Mf-positive index participants. First, the large difference in Mf prevalence between people with otherwise similar exposures (index cases compared to their household members) would be unlikely if reinfection was the main explanation. Even considering that index participants were significantly more likely than their household members to be adult males, who are known to be a high-risk group [9], their very high Mf prevalence suggests that reinfections were unlikely to be the only explanation. Second, a third of the 2019 Ag-positive/Mf-negative index participants were Mf-positive after 4.5 years. Although it is unknown whether they were initially Mf-positive prior to the 2018 MDA, these results suggest that any Mf clearance in these individuals resulting from the MDA were not sustained. This is consistent with recent findings from a Samoan cohort study which followed up Mf-positive individuals and found five out of eight of them to be Mf-positive 4.5 years later, despite receiving two rounds of directly observed treatment with IDA [25]. That study also reported that four out of 11 individuals who received directly observed treatment in 2023 were again Mf-positive in 2024, only 18 months later. Third, high rates of nonparticipation in the 2018 MDA is another possible explanation for the high-prevalence in index participants but is unlikely given that 94.5% of the Mf-positive participants reported taking MDA. Given there had been no significant change in community Ag and Mf prevalence between 2019 and 2023 [14], and based on the estimated Ag and Mf prevalence in the 22 villages where participants lived, ongoing transmission was likely to be occurring. Although new infections may be the explanation for some cases, the combination of the above observations strongly suggests recrudescence in at least some individuals.

Household members living with Mf-positive index cases were more likely to be Ag-positive and Mf-positive in 2023 compared to those who did not, and compared to members of the wider community. This points to the likely importance of household transmission or other shared risk factors. Regardless of the exact explanation for the higher Ag and Mf prevalence, our findings highlight the need to test and treat household members of known Ag-positive, and especially Mf-positive, cases.

One limitation of this study is that the reported MDA uptake by the index participants was based on self-reporting rather than observed or confirmed treatment. The 2019 survey occurred 7-9 months post-MDA and recollection bias, social desirability bias, or uncertainty could have affected the accuracy of participants’ responses. While the 94.5% MDA uptake reported amongst index participants may be higher than the actual MDA participation rates, it can reasonably be assumed that most of them did participate in the MDA in 2018, and it is highly likely that the uptake was above the 65% threshold recommended by WHO [10].

Our results raise concerns about the long-term effectiveness of single-dose triple-drug therapy for sustained clearance of Mf in Samoa. This study adds to the growing body of evidence [14,26] supporting the need for multiple rounds of triple-drug therapy for LF elimination in the Pacific Islands. Despite being proven effective for short-term clearance of Mf [11], long-term clearance has not been demonstrated in all areas of the Pacific. Furthermore, evidence-based clinical guidelines that support a proactive, targeted and consistent approach to treatment and follow-up of Ag-positive and Mf-positive people are required to prevent clinical disease and onward transmission, especially within households. These strategies will be required for achieving and sustaining elimination of LF as a public health problem in Samoa and elsewhere in the Pacific region.

## Ethical approval and informed consent

Ethics approvals were granted by the Samoan Ministry of Health and The University of Queensland Human Research Ethics Committee (protocol 2021/HE000895). The study was conducted in close collaboration with the Samoa Ministry of Health, the WHO country office in Samoa, and the Samoa Red Cross. Permission was sought from village leaders before entering a village. Verbal and written informed consent were obtained from all participants, or from the parents or guardians of participants under the age of 18 years.

## Data sharing

Data used in this article were collected during field surveys in Samoa. Communities in Samoa are small (some with less than 200 inhabitants), and sharing individual-level data that includes village and individual ages could enable identification of participants, violating the conditions of the study's ethics approval. We have therefore excluded this information from the data provided. Please contact the corresponding author if you would like access to the full dataset.

## Declarations of competing interest

The authors declare that they have no known competing financial interests or personal relationships that could have appeared to influence the work reported in this article.

## Appendix Supplementary materials

Image, application 1

Image, application 2
