# Supplementary material for: Progression of lymphatic filariasis antigenaemia and microfilaraemia over 4.5 years in antigen-positive individuals, Samoa 2019-2023
Source: Int J Infect Dis. 2025 Jun;155:None. doi: 10.1016/j.ijid.2025.107891 (PMC12069812; doi:10.1016/j.ijid.2025.107891)
Supplement: Supplementary file 1 [file mmc1.pdf]

International Journal of infectious diseases

Progression of lymphatic filariasis antigenaemia and microfilaraemia over 4.5 years in antigen-positive individuals, Samoa 2019-2023

Helen J. Mayfield [1,2], Benn Sartorius[1,2], Ramona Muttucumaru[1,2,3], Sarah Sheridan[1,2], Maddison Howlett [2], Beatris Mario Martin[1,2], Shannon M. Hedtke[4], Emma Field[3], Robert Thomson[5], Satupaitea Viali[6], Patricia M. Graves[7], Colleen L. Lau[1,2]*

* Corresponding author Colleen.Lau@UQ.edu.au

[1.] University of Queensland Centre for Clinical Research, Faculty of Health, Medicine, and Behavioural Sciences, The University of Queensland, Brisbane, Queensland, Australia

[2.] School of Public Health, Faculty of Health, Medicine, and Behavioural Sciences, The University of Queensland, Brisbane, Queensland, Australia

[3.] National Centre for Epidemiology and Population Health, The Australian National University, Canberra, Australia

[4.] Department of Environment and Genetics, La Trobe University, Bundoora, Victoria, Australia

[5.] Samoa Ministry of Health, Apia, Samoa

[6.] Oceania University of Medicine Samoa, Apia, Samoa

[7.] College of Public Health, Medical and Veterinary Sciences, James Cook University, Queensland, Australia

Ethics approvals were granted by the Samoan Ministry of Health and The University of Queensland Human Research Ethics Committee (protocol 2021/HE000895). Please contact the corresponding author if you would like access to the full dataset.

Table 1: Individual age group, sex and microfilaria (Mf) / antigen(Ag) status for participants in the Surveillance and Monitoring for the Elimination of Lymphatic Filariasis is Samoa (SaMELFS) 2023 follow-up survey. Group IDs are: A) Mf-positive index participants; B) Mf-negative index participants, C) household members of Mf-positive index participants; and D) all other household members

This data is provided for research transparency only.

Intellectual property remains with the authors.

Participant ID	Group	Age group 10 years	Sex	2019 Mf	2023 Ag	2023 Mf
1	A	50 to 59	Male	Positive	Positive	Positive
2	A	60 +	Female	Positive	Positive	Invalid
3	A	60 +	Male	Positive	Positive	Positive
4	A	30 to 39	Female	Positive	Positive	Positive
5	A	30 to 39	Female	Positive	Positive	Negative
6	A	40 to 49	Male	Positive	Positive	Positive
7	A	20 to 29	Male	Positive	Positive	Positive
8	A	40 to 49	Female	Positive	Positive	Positive
9	A	50 to 59	Male	Positive	Positive	Positive
10	A	50 to 59	Male	Positive	Positive	Positive
11	A	50 to 59	Male	Positive	Positive	Positive
12	A	60 +	Female	Positive	Positive	Negative
13	A	50 to 59	Male	Positive	Positive	Negative
14	A	50 to 59	Male	Positive	Positive	Negative
15	A	40 to 49	Male	Positive	Positive	Positive
16	A	40 to 49	Male	Positive	Positive	Invalid
17	A	60 +	Male	Positive	Positive	Positive
18	B	60 +	Female	Negative	Positive	Positive
19	B	50 to 59	Male	Negative	Positive	Positive
20	B	50 to 59	Male	Negative	Positive	Positive
21	B	60 +	Female	Negative	Positive	Positive
22	B	40 to 49	Female	Negative	Positive	Positive
23	B	50 to 59	Male	Negative	Positive	Positive
24	B	60 +	Male	Negative	Positive	Positive
25	B	60 +	Male	Negative	Positive	Positive
26	B	30 to 39	Female	Negative	Positive	Negative
27	B	10 to 19	Male	Negative	Positive	Negative
28	B	50 to 59	Female	Negative	Positive	Negative
29	B	30 to 39	Male	Negative	Positive	Negative
30	B	10 to 19	Female	Negative	Positive	Positive
31	B	50 to 59	Female	Negative	Positive	Negative
32	B	30 to 39	Male	Negative	Positive	Negative
33	B	40 to 49	Female	Negative	Positive	Negative
34	B	10 to 19	Female	Negative	Positive	Positive
35	B	10 to 19	Male	Negative	Positive	Positive
36	B	40 to 49	Female	Negative	Positive	Negative
37	B	60 +	Male	Negative	Positive	Positive
38	B	20 to 29	Female	Negative	Positive	Negative
39	B	30 to 39	Female	Negative	Positive	Negative
40	B	50 to 59	Male	Negative	Positive	Negative
41	B	20 to 29	Female	Negative	Positive	Negative
42	B	10 to 19	Female	Negative	Positive	Positive
43	B	50 to 59	Male	Negative	Positive	Positive
44	B	30 to 39	Male	Negative	Positive	Negative

45	B	40 to 49	Female	Negative	Positive	Positive
46	B	10 to 19	Female	Negative	Positive	Positive
47	B	40 to 49	Male	Negative	Positive	Negative
48	B	60 +	Female	Negative	Positive	Positive
49	B	50 to 59	Female	Negative	Negative	Negative
50	B	50 to 59	Male	Negative	Positive	Negative
51	B	50 to 59	Male	Negative	Positive	Negative
52	B	10 to 19	Male	Negative	Positive	Negative
53	B	50 to 59	Male	Negative	Positive	Positive
54	B	20 to 29	Male	Negative	Positive	Negative
55	B	30 to 39	Male	Negative	Positive	Negative
56	B	30 to 39	Male	Negative	Positive	Negative
57	B	20 to 29	Female	Negative	Positive	Negative
58	B	50 to 59	Male	Negative	Positive	Negative
59	B	20 to 29	Male	Negative	Positive	Positive
60	B	60 +	Male	Negative	Positive	Negative
61	B	40 to 49	Female	Negative	Positive	Negative
62	B	30 to 39	Male	Negative	Positive	Negative
63	B	40 to 49	Female	Negative	Positive	Negative
64	B	50 to 59	Male	Negative	Positive	Negative
65	B	20 to 29	Female	Negative	Positive	Negative
66	B	60 +	Male	Negative	Positive	Negative
67	B	10 to 19	Male	Negative	Positive	Negative
68	B	10 to 19	Male	Negative	Negative	Negative
69	B	30 to 39	Male	Negative	Negative	Negative
70	B	60 +	Male	Negative	Negative	Negative
71	B	30 to 39	Male	Negative	Negative	Negative
72	B	40 to 49	Male	Negative	Negative	Negative
73	B	60 +	Male	Negative	Negative	Negative
74	B	10 to 19	Male	Negative	Positive	Negative
75	B	40 to 49	Female	Negative	Negative	Negative
76	B	10 to 19	Male	Negative	Positive	Negative
77	B	60 +	Male	Negative	Negative	Negative
78	B	10 to 19	Male	Negative	Negative	Negative
79	B	30 to 39	Female	Negative	Positive	Negative
80	B	50 to 59	Male	Negative	Negative	Negative
81	B	30 to 39	Female	Negative	Positive	Negative
82	B	50 to 59	Male	Negative	Negative	Negative
83	B	50 to 59	Female	Negative	Negative	Negative
84	B	10 to 19	Male	Negative	Negative	Negative
85	C	5 to 9	Female	.	Negative	Negative
86	C	5 to 9	Male	.	Negative	Negative
87	C	5 to 9	Male	.	Negative	Negative
88	C	5 to 9	Female	.	Positive	Negative
89	C	5 to 9	Female	.	Negative	Negative
90	C	5 to 9	Male	.	Negative	Negative

91	C	5 to 9	Female	Negative	Positive	Negative
92	C	5 to 9	Male	.	Positive	Positive
93	C	5 to 9	Female	.	Negative	Negative
94	C	5 to 9	Female	.	Negative	Negative
95	C	5 to 9	Female	.	Positive	Negative
96	C	5 to 9	Female	.	Negative	Negative
97	C	5 to 9	Female	.	Positive	Negative
98	C	5 to 9	Female	.	Negative	Negative
99	C	5 to 9	Female	.	Negative	Negative
100	C	5 to 9	Male	.	Negative	Negative
101	C	10 to 19	Male	.	Negative	Negative
102	C	10 to 19	Female	.	Positive	Negative
103	C	10 to 19	Female	.	Negative	Negative
104	C	10 to 19	Male	.	Negative	Negative
105	C	10 to 19	Female	.	Negative	Negative
106	C	10 to 19	Female	.	Negative	Negative
107	C	10 to 19	Female	.	Negative	Negative
108	C	10 to 19	Female	.	Negative	Negative
109	C	10 to 19	Female	.	Negative	Negative
110	C	10 to 19	Male	.	Negative	Negative
111	C	10 to 19	Female	.	Positive	Negative
112	C	10 to 19	Female	.	Negative	Negative
113	C	10 to 19	Female	.	Negative	Negative
114	C	10 to 19	Male	.	Negative	Negative
115	C	10 to 19	Female	.	Negative	Negative
116	C	10 to 19	Female	.	Negative	Negative
117	C	20 to 29	Male	.	Positive	Negative
118	C	20 to 29	Female	.	Negative	Negative
119	C	20 to 29	Male	.	Negative	Negative
120	C	20 to 29	Male	.	Negative	Negative
121	C	20 to 29	Female	.	Negative	Negative
122	C	20 to 29	Female	.	Negative	Negative
123	C	30 to 39	Female	.	Negative	Negative
124	C	30 to 39	Male	.	Positive	Positive
125	C	30 to 39	Male	.	Negative	Negative
126	C	30 to 39	Female	.	Negative	Negative
127	C	30 to 39	Male	.	Negative	Negative
128	C	40 to 49	Female	.	Negative	Negative
129	C	40 to 49	Female	Negative	Negative	Negative
130	C	40 to 49	Female	.	Negative	Negative
131	C	40 to 49	Female	.	Negative	Negative
132	C	50 to 59	Male	Negative	Positive	Positive
133	C	50 to 59	Male	Negative	Positive	Positive
134	C	50 to 59	Male	.	Negative	Negative
135	C	50 to 59	Female	.	Negative	Negative
136	C	50 to 59	Female	.	Negative	Negative

137	C	50 to 59	Female	.	Negative	Negative
138	C	60 +	Female	Negative	Positive	Negative
139	C	60 +	Female	Negative	Positive	Positive
140	C	60 +	Female	.	Negative	Negative
141	C	60 +	Female	.	Negative	Negative
142	C	60 +	Female	.	Positive	Positive
143	C	60 +	Male	.	Negative	Negative
144	C	60 +	Male	Negative	Positive	Positive
145	D	30 to 39	Female	.	Positive	Negative
146	D	5 to 9	Male	.	Positive	Negative
147	D	10 to 19	Male	.	Positive	Negative
148	D	20 to 29	Male	.	Positive	Negative
149	D	10 to 19	Male	.	Positive	Negative
150	D	40 to 49	Female	.	Positive	Negative
151	D	10 to 19	Female	.	Negative	Negative
152	D	20 to 29	Male	.	Positive	Negative
153	D	10 to 19	Female	.	Positive	Negative
154	D	50 to 59	Female	.	Positive	Negative
155	D	20 to 29	Male	.	Positive	Negative
156	D	20 to 29	Male	.	Negative	Negative
157	D	30 to 39	Female	.	Positive	Negative
158	D	30 to 39	Female	.	Positive	Negative
159	D	20 to 29	Female	.	Negative	Negative
160	D	50 to 59	Female	.	Positive	Negative
161	D	40 to 49	Male	.	Positive	Negative
162	D	50 to 59	Male	.	Positive	Negative
163	D	40 to 49	Female	.	Positive	Negative
164	D	30 to 39	Female	.	Positive	Negative
165	D	40 to 49	Female	.	Positive	Negative
166	D	10 to 19	Female	.	Positive	Negative
167	D	5 to 9	Female	.	Positive	Negative
168	D	10 to 19	Female	.	Negative	Negative
169	D	30 to 39	Female	.	Positive	Negative
170	D	10 to 19	Female	.	Positive	Negative
171	D	10 to 19	Male	.	Positive	Negative
172	D	10 to 19	Male	.	Positive	Negative
173	D	10 to 19	Female	.	Positive	Negative
174	D	50 to 59	Male	.	Positive	Negative
175	D	10 to 19	Male	.	Positive	Positive
176	D	40 to 49	Male	.	Positive	Positive
177	D	30 to 39	Male	.	Positive	Positive
178	D	30 to 39	Female	.	Positive	Positive
179	D	50 to 59	Male	.	Positive	Positive
180	D	40 to 49	Male	.	Positive	Positive
181	D	40 to 49	Male	.	Positive	Positive
182	D	50 to 59	Male	.	Positive	Positive

183	D	5 to 9	Female	.	Negative	Negative
184	D	30 to 39	Female	.	Negative	Negative
185	D	40 to 49	Male	.	Negative	Negative
186	D	10 to 19	Female	.	Negative	Negative
187	D	30 to 39	Female	.	Negative	Negative
188	D	30 to 39	Female	.	Negative	Negative
189	D	40 to 49	Female	.	Negative	Negative
190	D	5 to 9	Female	.	Negative	Negative
191	D	60 +	Female	.	Negative	Negative
192	D	20 to 29	Male	.	Negative	Negative
193	D	5 to 9	Female	.	Negative	Negative
194	D	40 to 49	Female	.	Negative	Negative
195	D	20 to 29	Female	.	Negative	Negative
196	D	10 to 19	Male	.	Negative	Negative
197	D	10 to 19	Male	.	Negative	Negative
198	D	30 to 39	Female	.	Negative	Negative
199	D	40 to 49	Male	.	Negative	Negative
200	D	10 to 19	Female	.	Negative	Negative
201	D	5 to 9	Female	.	Negative	Negative
202	D	50 to 59	Female	.	Negative	Negative
203	D	60 +	Male	.	Negative	Negative
204	D	5 to 9	Female	.	Negative	Negative
205	D	30 to 39	Female	.	Negative	Negative
206	D	10 to 19	Male	.	Negative	Negative
207	D	10 to 19	Male	.	Negative	Negative
208	D	10 to 19	Male	.	Negative	Negative
209	D	10 to 19	Male	.	Negative	Negative
210	D	20 to 29	Female	.	Negative	Negative
211	D	50 to 59	Female	.	Negative	Negative
212	D	10 to 19	Male	.	Negative	Negative
213	D	50 to 59	Male	.	Negative	Negative
214	D	10 to 19	Male	.	Negative	Negative
215	D	40 to 49	Female	.	Negative	Negative
216	D	40 to 49	Male	.	Negative	Negative
217	D	10 to 19	Male	.	Negative	Negative
218	D	5 to 9	Male	.	Negative	Negative
219	D	10 to 19	Female	.	Negative	Negative
220	D	10 to 19	Female	.	Negative	Negative
221	D	10 to 19	Female	.	Negative	Negative
222	D	5 to 9	Female	.	Negative	Negative
223	D	10 to 19	Female	.	Negative	Negative
224	D	10 to 19	Female	.	Negative	Negative
225	D	10 to 19	Male	.	Negative	Negative
226	D	10 to 19	Female	.	Negative	Negative
227	D	30 to 39	Female	.	Negative	Negative
228	D	10 to 19	Male	.	Negative	Negative

229	D	10 to 19	Male	.	Negative	Negative
230	D	20 to 29	Female	.	Negative	Negative
231	D	20 to 29	Female	.	Negative	Invalid
232	D	10 to 19	Male	.	Negative	Negative
233	D	5 to 9	Male	.	Negative	Negative
234	D	50 to 59	Female	.	Negative	Negative
235	D	10 to 19	Female	.	Negative	Negative
236	D	5 to 9	Male	.	Negative	Negative
237	D	5 to 9	Female	.	Negative	Negative
238	D	10 to 19	Female	.	Negative	Negative
239	D	20 to 29	Male	.	Negative	Negative
240	D	30 to 39	Female	.	Negative	Negative
241	D	10 to 19	Male	.	Negative	Negative
242	D	10 to 19	Female	.	Negative	Negative
243	D	20 to 29	Male	.	Negative	Negative
244	D	30 to 39	Female	.	Negative	Negative
245	D	10 to 19	Male	.	Negative	Negative
246	D	20 to 29	Female	.	Negative	Negative
247	D	20 to 29	Female	.	Negative	Negative
248	D	10 to 19	Male	.	Negative	Negative
249	D	5 to 9	Male	.	Negative	Negative
250	D	5 to 9	Female	.	Negative	Negative
251	D	5 to 9	Male	.	Negative	Negative
252	D	50 to 59	Female	.	Negative	Negative
253	D	10 to 19	Male	.	Negative	Negative
254	D	10 to 19	Male	.	Negative	Negative
255	D	5 to 9	Male	.	Negative	Negative
256	D	5 to 9	Female	.	Negative	Negative
257	D	30 to 39	Female	.	Negative	Negative
258	D	50 to 59	Male	.	Negative	Negative
259	D	10 to 19	Male	.	Negative	Negative
260	D	5 to 9	Male	.	Negative	Negative
261	D	5 to 9	Female	.	Negative	Negative
262	D	10 to 19	Male	.	Negative	Negative
263	D	10 to 19	Female	.	Negative	Negative
264	D	10 to 19	Male	.	Negative	Negative
265	D	10 to 19	Female	.	Negative	Negative
266	D	10 to 19	Female	.	Negative	Negative
267	D	5 to 9	Female	.	Negative	Negative
268	D	5 to 9	Female	.	Negative	Negative
269	D	60 +	Female	.	Negative	Negative
270	D	10 to 19	Male	.	Negative	Negative
271	D	5 to 9	Female	.	Negative	Negative
272	D	10 to 19	Male	.	Negative	Negative
273	D	10 to 19	Female	.	Negative	Negative
274	D	30 to 39	Female	.	Negative	Negative

275	D	10 to 19	Male	.	Negative	Negative
276	D	10 to 19	Female	.	Negative	Negative
277	D	10 to 19	Male	.	Negative	Negative
278	D	10 to 19	Female	.	Negative	Negative
279	D	40 to 49	Female	.	Negative	Negative
280	D	10 to 19	Female	.	Negative	Negative
281	D	10 to 19	Male	.	Negative	Negative
282	D	60 +	Male	.	Negative	Negative
283	D	5 to 9	Female	.	Negative	Negative
284	D	30 to 39	Female	.	Negative	Negative
285	D	10 to 19	Male	.	Negative	Negative
286	D	40 to 49	Female	.	Negative	Negative
287	D	10 to 19	Female	.	Negative	Negative
288	D	20 to 29	Female	.	Negative	Negative
289	D	60 +	Female	.	Negative	Negative
290	D	20 to 29	Female	.	Negative	Negative
291	D	5 to 9	Male	.	Negative	Negative
292	D	10 to 19	Female	.	Negative	Negative
293	D	20 to 29	Female	.	Negative	Negative
294	D	5 to 9	Male	.	Negative	Negative
295	D	10 to 19	Female	.	Negative	Negative
296	D	5 to 9	Female	.	Negative	Negative
297	D	60 +	Female	.	Negative	Negative
298	D	5 to 9	Female	.	Negative	Negative
299	D	5 to 9	Female	.	Negative	Negative
300	D	5 to 9	Female	.	Negative	Negative
301	D	10 to 19	Male	.	Negative	Negative
302	D	10 to 19	Female	.	Negative	Negative
303	D	40 to 49	Female	.	Negative	Negative
304	D	10 to 19	Female	.	Negative	Negative
305	D	10 to 19	Male	.	Negative	Negative
306	D	5 to 9	Male	.	Negative	Negative
307	D	10 to 19	Male	.	Negative	Negative
308	D	10 to 19	Male	.	Negative	Negative
309	D	20 to 29	Male	.	Negative	Negative
310	D	5 to 9	Female	.	Negative	Negative
311	D	10 to 19	Female	.	Negative	Negative
312	D	5 to 9	Female	.	Negative	Negative
313	D	10 to 19	Female	.	Negative	Negative
314	D	30 to 39	Male	.	Negative	Negative
315	D	5 to 9	Male	.	Negative	Negative
316	D	10 to 19	Female	.	Negative	Negative
317	D	10 to 19	Male	.	Negative	Negative
318	D	10 to 19	Female	.	Negative	Negative
319	D	10 to 19	Female	.	Negative	Negative
320	D	60 +	Male	.	Negative	Negative

321	D	30 to 39	Female	.	Negative	Negative
322	D	20 to 29	Female	.	Negative	Negative
323	D	20 to 29	Female	.	Negative	Negative
324	D	5 to 9	Male	.	Negative	Negative
325	D	10 to 19	Male	.	Negative	Negative
326	D	5 to 9	Male	.	Negative	Negative
327	D	5 to 9	Male	.	Negative	Negative
328	D	40 to 49	Female	.	Negative	Negative
329	D	5 to 9	Female	.	Negative	Negative
330	D	10 to 19	Male	.	Negative	Negative
331	D	30 to 39	Female	.	Negative	Negative
332	D	10 to 19	Female	.	Negative	Negative
333	D	20 to 29	Male	.	Negative	Negative
334	D	30 to 39	Female	.	Negative	Negative
335	D	10 to 19	Male	.	Negative	Negative
336	D	10 to 19	Female	.	Negative	Negative
337	D	5 to 9	Female	.	Negative	Negative
338	D	5 to 9	Male	.	Negative	Negative
339	D	10 to 19	Male	.	Negative	Negative
340	D	30 to 39	Female	.	Negative	Negative
341	D	10 to 19	Male	.	Negative	Negative
342	D	10 to 19	Male	.	Negative	Negative
343	D	20 to 29	Female	.	Negative	Negative
344	D	50 to 59	Male	.	Negative	Negative
345	D	40 to 49	Female	.	Negative	Negative
346	D	10 to 19	Female	.	Negative	Negative
347	D	20 to 29	Female	.	Negative	Negative
348	D	60 +	Female	.	Negative	Negative
349	D	50 to 59	Female	.	Negative	Negative
350	D	10 to 19	Male	.	Negative	Negative
351	D	5 to 9	Male	.	Negative	Negative
352	D	30 to 39	Female	.	Negative	Negative
353	D	10 to 19	Female	.	Negative	Negative
354	D	10 to 19	Female	.	Negative	Negative
355	D	5 to 9	Male	.	Negative	Negative
356	D	20 to 29	Female	.	Negative	Negative
357	D	20 to 29	Female	.	Negative	Negative
358	D	10 to 19	Female	.	Negative	Negative
359	D	5 to 9	Male	.	Negative	Negative
360	D	40 to 49	Female	.	Negative	Negative
361	D	5 to 9	Male	.	Negative	Negative
362	D	10 to 19	Male	.	Negative	Negative
363	D	5 to 9	Female	.	Negative	Negative
364	D	40 to 49	Female	.	Negative	Negative
365	D	20 to 29	Female	.	Negative	Negative
366	D	40 to 49	Female	.	Negative	Negative

367	D	10 to 19	Male	.	Negative	Negative
368	D	10 to 19	Male	.	Negative	Negative
369	D	5 to 9	Male	.	Negative	Negative
370	D	10 to 19	Female	.	Negative	Negative
371	D	5 to 9	Female	.	Positive	Negative
372	D	10 to 19	Male	.	Positive	Negative
373	D	30 to 39	Male	.	Positive	Positive
374	D	10 to 19	Male	.	Negative	Negative
375	D	5 to 9	Female	.	Negative	Negative
376	D	60 +	Female	.	Negative	Negative
377	D	50 to 59	Female	.	Negative	Negative
378	D	20 to 29	Female	.	Negative	Negative
379	D	10 to 19	Female	.	Negative	Negative
