# Supplementary material for: Progression of lymphatic filariasis antigenaemia and microfilaraemia over 4.5 years in antigen-positive individuals, Samoa 2019-2023
Source: Int J Infect Dis. 2025 Jun;155:None. doi: 10.1016/j.ijid.2025.107891 (PMC12069812; doi:10.1016/j.ijid.2025.107891)
Supplement: Supplementary file 2 [file mmc2.pdf]

Progression of lymphatic filariasis antigenaemia and microfilaraemia over 4.5 years in antigen-positive individuals, Samoa 2019-2023

Helen J. Mayfield, Benn Sartorius, Ramona Muttucumaru, Sarah Sheridan, Maddison Howlett, Beatris Mario Martin, Shannon M. Hedtke, Emma Field, Robert Thomson, Satupaitea Viali, Patricia M. Graves, Colleen L. Lau

<u>Supplementary material</u>

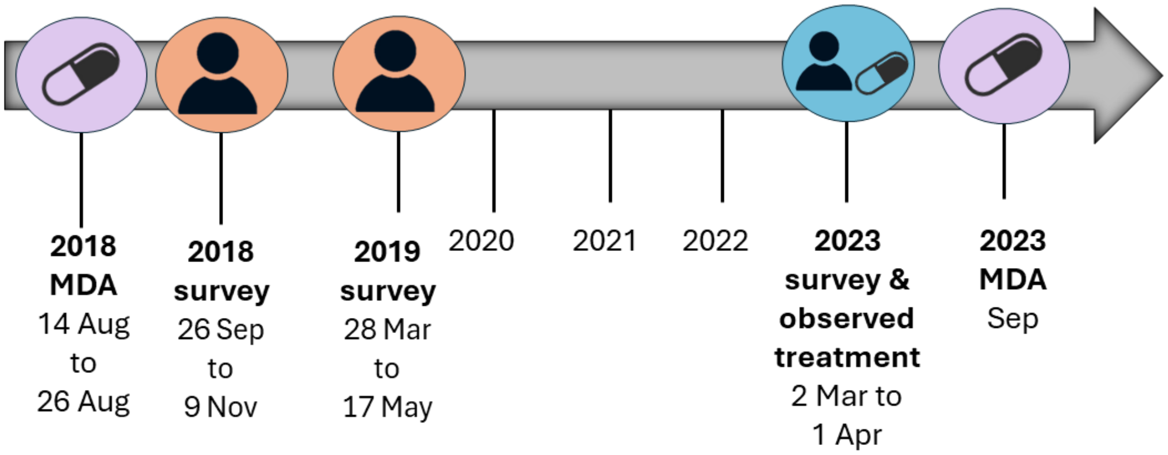

Supplementary Figure S1: Timeline of Surveillance and Monitoring to Eliminate Lymphatic Filariasis in Samoa (SaMELFS) surveys relative to national mass drug administrations (MDA)

Supplementary Table S1: Summary of index participant distribution in the multi-index households in the 2023 follow-up survey in Samoa

Index participant category	Number of houses surveyed	Number of index participants
2019 Mf-positive index	15	1
	1	2
2019 Mf-negative index	53	1
	9	2
	1	3

Supplementary Table S2: Group allocations for index participants from multi-index households in the 2023 follow-up survey in Samoa, allocated based on the presence of a 2019 microfilaria-positive (Mf+) index participant. Group A = 2019 Mf+ index participants, Group B = 2019 Mf-negative index participants, and Group C1 = Household members of group A who were also 2019 Mf-negative index participants.

Multi-index households	Total index participants in household (n)	2019 Mf-positive index		2019 Mf-negative index	
		Participants (n)	Group	Participants (n)	Group
1	2	1	A	1	C1
2	2	1	A	1	C1
3	3	1	A	2	C1
4	3	1	A	2	C1
5	2	1	A	1	C1
6	2	0	.	2	B
7	2	0	.	2	B
8	2	0	.	2	B
9	2	0	.	2	B
10	2	0	.	2	B
11	2	0	.	2	B
12	2	0	.	2	B
13	2	0	.	2	B
14	3	0	.	3	B

Supplementary Table S3: Odds ratios (OR) for Ag-positivity and Mf-positivity in index participants compared to household members. Group A = 2019 Mf-positive index participants, Group B = 2019 Mf-negative index participants, and Group C = Household members of Group A, Group D = Household members of Group B

Comparison	Antigen		Microfilaria	
	**Odds Ratio OR (95% CIs)**	**P-value**	**Odds Ratio OR (95% CIs)**	**P-value**
Group A vs Group C	N/A	<0.001	11.4 (0.09- 150.2)	0.064
Group B vs Group D	21.0 (8.6-51.3)	<0.001	7.3 (2.4-22.7)	0.001
Group A&B vs Group C&D	26.6 (10.9 – 64.6)	<0.001	8.0 (2.8-22.7)	<0.001

Supplementary Table S4: Antigen (Ag) and microfilaria (Mf) crude prevalence for 2019 Ag-positive index participants and their household members in the 2023 SaMELFS follow-up survey.

		Prevalence in index participants % (95% CIs)	Prevalence in household members % (95% CIs)
Mf-positive index in 2019	Ag	100.0 (78.3-100)	25.0 (15.7-37.3)
	Mf	64.7 (41.2-82.8)	11.7 (5.5-22.5)
Mf-negative index in 2019	Ag	79.1 (67.8-87.2)	15.7 (11.6-21.0)
	Mf	28.4 (18.9-40.2)	3.8 (1.9-7.2)

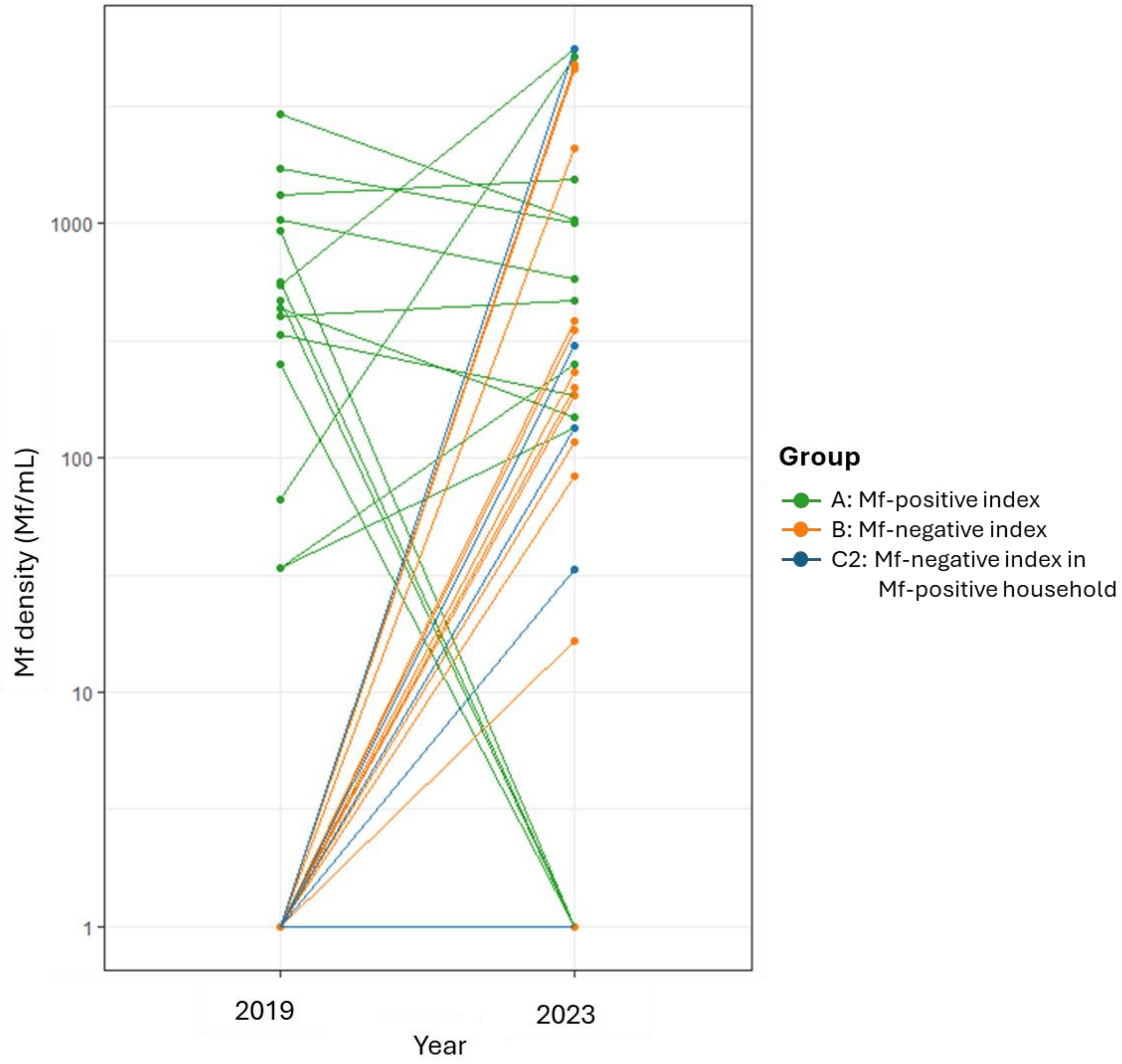

Supplementary Figure S2: Change in individual microfilaria (Mf) geometric mean density (Mf/mL) from 2019 to 2023 for index participants with Mf results in both years in the 2023 follow-up study in Samoa. Note log scale on Y axis.
